# Association Between Attitude Toward a Healthy Lifestyle, Lifestyle Behaviors, Sociodemographic Characteristics, and Body Mass Index: A Cross-Sectional Study

**DOI:** 10.3390/nu18030500

**Published:** 2026-02-02

**Authors:** Marija Ljubičić, Tamara Sorić, Ivana Gusar, Donata Vidaković Samaržija, Gordana Ivković, Ana Pejdo, Jelena Vučak Lončar, Mira Klarin, Nita Šarić, Ivana Kolčić

**Affiliations:** 1 Department of Health Studies, University of Zadar, Splitska 1, 23000 Zadar, Croatia; igusar@unizd.hr; 2 Psychiatric Hospital Ugljan, Otočkih Dragovoljaca 42, 23275 Ugljan, Croatia; tamara.novoselic@pbu.hr; 3 Department of Teacher and Preschool Teacher Education, University of Zadar, Franje Tuđmana 24i, 23000 Zadar, Croatia; dovidak@unizd.hr (D.V.S.); mklarin@unizd.hr (M.K.); 4 Centre for Gymnastics and Student Sports, University of Zadar, Trg Kneza Višeslava 9, 23000 Zadar, Croatia; givkovic@unizd.hr; 5 Department of Geography, University of Zadar, Franje Tuđmana 24i, 23000 Zadar, Croatia; apejdo@unizd.hr; 6 Endocrinology Department, General Hospital Zadar, Bože Peričića 5, 23000 Zadar, Croatia; jelena.vucak.loncar@bolnica-zadar.hr; 7 Agricultural, Food and Veterinary School Stanko Ožanić, Franje Tuđmana 24/h, 23000 Zadar, Croatia; sarichnita@gmail.com; 8 Andrija Stampar Teaching Institute of Public Health, Mirogojska 16, 10000 Zagreb, Croatia; ivana.kolcic@stampar.hr; 9 School of Medicine, University of Split, Šoltanska 2A, 21000 Split, Croatia; 10 Croatian Lifestyle Medicine Association, Nova cesta 136, 10000 Zagreb, Croatia

**Keywords:** attitude, healthy lifestyle, body mass index, lifestyle behavior, self-rated health

## Abstract

**Background/Objectives**: Healthy lifestyle behaviors are major contributors to overall health and disease prevention. This cross-sectional study aimed to assess attitude toward a healthy lifestyle and its associations with lifestyle behaviors, body mass index (BMI), excess body weight, sociodemographic characteristics, and self-rated health in adults. **Methods**: The Attitudes toward a Healthy Lifestyle Questionnaire was administered to 570 Croatian adults between November and December 2025. Multiple linear and binary logistic regression models were used to examine associations between lifestyle behaviors (number of daily meals, sitting time, sleep duration), smoking and e-cigarette use, alcohol consumption, BMI, self-rated health, and attitude toward a healthy lifestyle. **Results**: The median attitude toward a healthy lifestyle score was 52.0 (IQR = 10), corresponding to 62% of the maximum possible score. A more positive attitude toward a healthy lifestyle was associated with a higher number of daily meals (β = 0.16, *p* = 0.001), longer sleep duration (β = 0.17, *p* < 0.001), lower sitting time (β = −0.11, *p* = 0.010), and lower BMI (β = −0.24, *p* < 0.001). Smoking was negatively associated with attitude toward a healthy lifestyle (β = −0.18; *p* < 0.001), while e-cigarette use was associated with fewer daily meals (β = −0.10; *p* = 0.025). Longer sleep duration increased the odds of excellent self-rated health (OR = 1.31, *p* = 0.014), and a more positive attitude toward a healthy lifestyle was associated with lower odds of excess body weight (OR = 0.92, *p* < 0.001). **Conclusions**: Attitude toward a healthy lifestyle is significantly associated with lifestyle behaviors, BMI, excess body weight, and self-rated health, highlighting the importance of psychological factors in promoting sustainable healthy lifestyles.

## 1. Introduction

Lifestyle behaviors are major determinants of population health and well-being [1]. Chronic non-communicable diseases (NCDs) account for more than 80% of global mortality and are closely linked to modifiable lifestyle-related risk factors, including physical inactivity, unhealthy dietary patterns, excess body weight, and tobacco and alcohol use [2,3]. Projections indicate that by 2030, nearly 500 million additional people worldwide will be living with obesity, diabetes, cardiovascular disease, or other lifestyle-associated conditions. In Croatia, more than one third of adults (37%) report having at least one chronic condition, a prevalence comparable to the European Union average (36%), underscoring the public health relevance of lifestyle-related risk factors in this population [4]. As emphasized by the World Health Organization (WHO), health encompasses not only the absence of disease but also physical, mental, and social well-being; therefore, adopting healthy lifestyle behaviors is essential for sustaining health across the lifespan [1,5].

Lifestyle represents a multidimensional construct consisting of interrelated behaviors that influence daily functioning, such as diet, physical activity, sleep, social engagement, mental well-being, stress regulation, and the avoidance of harmful behaviors, including smoking and excessive alcohol consumption [6,7]. While a healthy lifestyle is widely acknowledged as a key determinant of disease prevention and recovery, its importance is often underestimated in the absence of immediate health concerns [7]. Individuals typically consider lifestyle changes only when health begins to deteriorate; however, modifying long-established habits is challenging and requires substantial motivation and support [8]. Nevertheless, lifestyle remains one of the most controllable and impactful determinants of long-term health [9].

Contemporary lifestyles are frequently characterized by suboptimal dietary habits, insufficient physical activity, inadequate sleep, and chronic stress. These behaviors interact to influence metabolic health, cognitive functioning, systemic inflammation, and body weight status [10,11,12]. Diets high in ultra-processed foods, irregular meal patterns, and breakfast skipping have been associated with an increased risk of obesity and metabolic disturbances [13]. Importantly, body weight and body mass index (BMI) are shaped by a complex interplay of dietary and nutritional factors, including overall energy intake, macronutrient composition, portion size, meal timing, and broader dietary patterns such as higher intake of fruits, vegetables, whole grains, and lean proteins, which have been associated with more favorable weight outcomes [14]. Physical inactivity and poor sleep further contribute to metabolic dysfunction, weight gain, impaired mental well-being, and reduced quality of life [10,11,12]. In addition, the quality of interpersonal relationships influences the development of chronic conditions beyond what can be fully explained by socioeconomic or behavioral determinants alone [15].

Although lifestyle is a broad and multidimensional construct, specific behavioral indicators provide practical and measurable ways to assess everyday lifestyle patterns at the population level. Indicators such as number of daily meals, sitting time, sleep duration, smoking, and alcohol consumption capture habitual behaviors that are strongly linked to health and well-being. Previous studies have shown that dietary habits, meal frequency, sedentary behavior, physical activity, sleep duration, smoking, and alcohol consumption are associated with better well-being and health-related outcomes, even after controlling for sociodemographic characteristics and chronic conditions [16,17].

Although lifestyle strongly contributes to disease development, genetic, psychosocial, and environmental factors also play important roles in shaping individual health outcomes [18,19,20]. Health is further influenced by broader social determinants, including living environment, socioeconomic position, education, and social roles [21]. From a behavioral and social–ecological perspective, sociodemographic characteristics shape access to health-related resources, perceived behavioral control, and normative expectations, thereby influencing the formation and maintenance of lifestyle attitudes [22].

Sociodemographic characteristics such as age, sex, and education are often associated with differences in lifestyle attitudes and behaviors [21,23,24,25,26]. Younger individuals tend to be more inclined toward risky lifestyle behaviors, whereas older adults often exhibit more stable behaviors [25,27,28,29]. Women and men differ in health priorities, perceptions of nutrition and physical activity, and susceptibility to weight gain across the lifespan [23,24,26,30]. Health conditions also vary between men and women and tend to occur at different stages of life [21,31]. Higher educational level is associated with health literacy and engagement in health-promoting behaviors, whereas lower levels of education are often linked to poorer health outcomes, higher stress levels, and reduced self-esteem [21,32]. Furthermore, caregiving responsibilities represent social role-related factors that may adversely affect lifestyle behaviors, as caregivers frequently experience increased stress, disrupted sleep, reduced physical activity, and poorer dietary patterns [33,34,35]. Considering these factors is therefore essential for interpreting population-level differences in lifestyle attitudes.

Attitudes are defined as psychological evaluations of concepts or behaviors that guide decision-making [36]. They play a central role in shaping health-related behaviors, thereby influencing health outcomes, healthcare costs, and long-term disease management [37]. However, attitudes are often long-standing, stable, and resistant to change, which may hinder the adoption and maintenance of health-promoting behaviors and facilitate the persistence of preventable health risks [36].

Despite the recognized importance of lifestyle attitudes, research specifically examining attitudes toward a healthy lifestyle remains limited. Existing studies have primarily focused on observable health behaviors or on individuals with diagnosed conditions, particularly in the context of disease management, while the preventive role of lifestyle attitudes in the general population remains insufficiently explored [38,39]. Moreover, the links between lifestyle attitudes, sociodemographic characteristics, everyday behavioral indicators, and health-related outcomes have rarely been examined simultaneously at the population level [30]. Similarly, the effects of small and realistic lifestyle changes, such as meal frequency, sedentary behavior, and sleep, remain unclear [40].

Previous research has demonstrated that health behaviors, motivation for health behavior, lifestyle attitudes, emotional well-being, and higher BMI are interrelated and linked to adverse health outcomes [19,27,38,39,41,42]. The theory of planned behavior provides a useful framework for understanding these relationships, suggesting that behavior is influenced by attitudes, subjective norms, and perceived behavioral control [43]. Behavioral beliefs shape attitudes toward behavior, normative beliefs shape perceived social pressure, and control beliefs shape perceived behavioral control or self-efficacy, which may also moderate the relationship between attitudes and intentions [43]. Similarly, the health belief model emphasizes that beliefs, perceived health threats, and confidence in one’s ability to act are central to shaping preventive health behaviors [30,44].

There remains a lack of studies examining population-level attitudes toward healthy lifestyles and their associations with dietary habits, sedentary behavior, sleep patterns, and body weight. Most existing research continues to prioritize observable behaviors rather than directly assessing underlying attitudes that may drive them. Consequently, current findings often reflect behavioral outcomes rather than enduring psychological orientations toward health, underscoring the need for research that integrates attitudes with lifestyle behaviors and health indicators.

Given the multifactorial nature of lifestyle and its impact on health, this study aimed to assess attitude toward a healthy lifestyle and to examine its associations with selected lifestyle behaviors, sociodemographic characteristics, BMI, excess body weight, and self-rated health in a community-based adult population. This study focuses on practical, measurable behavioral indicators, including number of daily meals, sitting time, and sleep duration, as reliable proxies for everyday lifestyle patterns. The present study hypothesized that attitude toward a healthy lifestyle would be significantly associated with lifestyle behaviors (number of daily meals, sitting time, and sleep duration), BMI, excess body weight, and self-rated health, and additionally explored whether these associations differ across sociodemographic characteristics. A comprehensive understanding of these relationships may inform the development of targeted public health interventions aimed at promoting sustainable healthy lifestyles and preventing chronic disease.

## 2. Materials and Methods

### 2.1. Participants

A cross-sectional study was conducted during November and December 2025 using a non-random convenience sample of adults residing in Croatia. Participants were recruited through online platforms, including email, Facebook, LinkedIn, and WhatsApp. Data were collected using an anonymous online questionnaire administered via Google Forms. Information on the total number of individuals who received the study invitation was not available; therefore, the response rate could not be calculated.

A total of 576 individuals accessed and completed the questionnaire. Eligibility criteria included being 18 years of age or older, residing in Croatia, and voluntarily agreeing to participate in the study. Exclusion criteria were tourist status, international student status, and incomplete questionnaire responses. Based on these criteria, six participants were excluded, resulting in a final analytic sample of 570 participants.

Before providing informed consent, participants received detailed information about the purpose and aims of the study and the voluntary nature of participation. The principal investigator’s contact information was provided to allow participants to ask questions or request additional information prior to participation. Informed consent was obtained electronically by clicking a consent button before accessing the questionnaire. Participation was entirely voluntary, and no financial or material incentives were offered.

Sample size adequacy was determined using an online sample size calculator [45]. Based on the population of Croatia (approximately 3.87 million inhabitants), a 95% confidence level, and a margin of error of ±5%, the minimum required sample size was estimated to be 385 participants.

The study protocol was approved by the Ethics Committee of the University of Zadar, Croatia (approval number: 2198-1-79-62-25-08) and was conducted in accordance with the ethical principles of the Declaration of Helsinki and its revisions. The study was registered in the ClinicalTrials.gov (ID: NCT07309484).

### 2.2. Questionnaire

Data were collected using a structured, self-administered questionnaire developed for the purposes of this study and administered in the Croatian language. The questionnaire consisted of two main parts.

The first part collected information on participants’ sociodemographic and general characteristics, including sex, age, marital status, level of education, place of residence, employment status, type of work, and economic status. Participants were also asked about caregiving responsibilities for vulnerable family members, including caring for a child with a disability, a child with a chronic disease, an ill adult family member, and an older person.

Health-related variables included self-reported body weight and height, self-rated health status, average daily sleep duration (hours per day), daily sitting time (hours per day) and meal frequency (number of meals consumed per day). Negative lifestyle habits were assessed using self-reported questions on cigarette smoking, e-cigarette use, and alcohol consumption. For each negative behavior, participants indicated whether they engaged in each behavior (no, yes, or occasionally). Self-reported body weight and height were used to calculate BMI using the standard formula (BMI = kg/m^2^) [46]. Although self-reported anthropometric data may be subject to reporting bias, this approach is commonly used in population-based studies [47]. Excess body weight was defined as BMI ≥ 25.0 kg/m^2^ in accordance with WHO criteria [48]. Excellent self-rated health was defined as participants’ self-assessment of their overall health as “excellent” on a standardized health rating scale. Self-rated health is a commonly used indicator of overall health status in population health research and has been shown to be a valid and reliable measure in large surveys [49,50,51].

The second part of the questionnaire consisted of the Attitudes Toward a Healthy Lifestyle Questionnaire, designed to assess individuals’ attitudes toward healthy habits and motivation across the six core pillars of lifestyle medicine: proper nutrition, regular physical activity, stress management, sleep quality, interpersonal relationships, and avoidance of negative lifestyle habits (smoking and alcohol consumption). The questionnaire comprises 15 items grouped into seven domains: nutrition (items 7 and 8), physical activity (item 9), sleep (item 6), stress (items 4 and 11), interpersonal relationships (items 10, 13, and 14), harmful habits (items 2 and 15), and healthy habits and motivation (items 1, 3, 5, and 12). Each item was rated on a 5-point Likert scale ranging from 1 (strongly disagree) to 5 (strongly agree). Reverse-worded items (items 1, 4, 7, 8, 10, and 12) were scored inversely. A total attitude score was calculated by summing responses to all items, yielding a possible score range from 15 to 75, with higher scores indicating more positive attitude toward a healthy lifestyle. The full questionnaire, including all 15 items, response options, and scoring, is provided in Supplementary Table S1.

Content validity was evaluated prior to data collection using the Content Validity Index (CVI) by a panel of five experts with several years of professional experience in public health, nutrition, and biomedicine. For this validation process, each item was rated for relevance on a 4-point scale (1 = not relevant to 4 = highly relevant). Items with an item-level CVI (I-CVI) ≥ 0.78 were considered acceptable, while items below this threshold were subject to revision. All items demonstrated high relevance (I-CVI ≥ 0.80). The scale-level CVI, calculated as the average of the I-CVI values (S-CVI/Ave), was 0.89, indicating good overall content validity. The average modified kappa coefficient was 0.72, reflecting substantial agreement beyond chance [52]. Cronbach’s alpha was α = 0.69, reflecting acceptable internal consistency for a multidimensional, exploratory instrument encompassing conceptually diverse domains.

### 2.3. Statistical Analysis

Statistical analyses were performed using JASP software, version 0.95.4 (JASP Team, University of Amsterdam, Amsterdam, The Netherlands). A significance level of *p* < 0.05 was applied throughout. The normality of data distributions was assessed using the Kolmogorov–Smirnov test.

Categorical variables are presented as absolute and relative frequencies, and differences between them were examined using the Chi-square test. Continuous variables are reported as medians (Mdn) and interquartile ranges (IQR) due to non-normally distributed data. Associations between variables were assessed using Spearman’s rank correlation coefficient. Partial correlations based on ranked data (Spearman) were performed to account for non-normal distributions and were adjusted for age, sex, employment status, type of work, and educational level. Differences between independent groups were examined using the Kruskal-Wallis test or the Mann-Whitney U test, as appropriate. The reliability of the scale was assessed using Cronbach’s alpha coefficient.

Multiple linear regression models were performed to examine associations between lifestyle-related outcomes and their predictors. Separate regression models were constructed for each outcome variable, including attitude toward a healthy lifestyle, number of daily meals, sleep duration, sitting time, and BMI. Predictor variables included sociodemographic characteristics, caregiving responsibilities, lifestyle behaviors (smoking, e-cigarette use, and alcohol consumption), and self-rated health. All predictors were entered simultaneously into each model to assess independent associations and reduce potential confounding. Multicollinearity among predictors was checked. Participants with incomplete data were excluded from the analyses, so that no missing values were present in the regression models. Regression coefficients (β), odds ratios (OR) with 95% confidence intervals (CI), and *p*-values were reported.

An a priori power analysis was conducted using G*Power software version 3.1.9.7. (Universität Kiel and University Düsseldorf, Düsseldorf, Germany) to ensure sufficient statistical power for the multiple linear regression analyses. Based on 21 predictors, an effect size of f^2^ = 0.15, a desired power of 1 − β = 0.95, and a significance level of α = 0.05, the minimum required sample size was estimated to be 226 participants. A post hoc power analysis based on the final sample demonstrated satisfactory test power (1 − β = 0.99).

To identify independent predictors of excellent self-rated health and excess body weight, binary logistic regression analyses were conducted. Two binary outcome variables were defined: excess body weight (BMI ≥ 25.0 kg/m^2^) and excellent self-rated health. Separate logistic regression models were constructed for each outcome. Independent variables included sociodemographic factors, caregiving responsibilities, lifestyle behaviors (cigarette and e-cigarette use and alcohol consumption), number of daily meals, sitting time, sleep duration, BMI, and overall attitude toward a healthy lifestyle. OR with 95% CI and *p*-values were reported, with OR > 1 indicating increased odds and OR < 1 indicating decreased odds of the outcome.

An a priori power analysis for the logistic regression models was conducted using G*Power software. Assuming an OR of 1.5 for the key predictor sex in relation to excellent self-rated health, with a significance level of α = 0.05 and a desired power of 1 − β = 0.95, the minimum required sample size was estimated to be 503 participants. A post hoc power analysis based on the final sample indicated adequate power (1 − β = 0.97) to detect meaningful effects for this predictor.

## 3. Results

### 3.1. Sociodemographic Characteristics of Participants

The median age of participants was 44.0 years (IQR = 15.0), with the most represented age group being 31–45 years (40.7%). The majority of participants were women (83.9%) and resided in urban areas (75.4%). Most participants were married or living in a domestic partnership (61.8%) and had children (61.9%; median number of children = 2.0). Regarding education and employment, most participants had completed a master’s degree (43.5%) and were employed (82.8%). Participants most commonly reported working in jobs involving minimal physical activity and high levels of interpersonal contact (36.8%) or in intensive work requiring constant interaction with people (21.9%). A smaller proportion of participants reported caregiving responsibilities for family members, including caring for a child with a disability (4.0%), a child with a chronic disease (3.9%), an ill adult family member (9.5%), or an older person (12.1%) (Supplementary Table S2).

### 3.2. Attitude Toward a Healthy Lifestyle and Lifestyle Characteristics of Participants

The median score for attitude toward a healthy lifestyle was 52.0 (IQR = 10.0) on a scale ranging from 15 to 75, corresponding to approximately 62% of the maximum possible score and indicating a moderate to slightly above-average positive attitude. Participants reported a median daily sitting time of 5.0 (IQR = 4.0) hours, a median of three meals per day, and a median sleep duration of 7.0 (IQR = 2.0) hours per day.

The median body weight and height were 73.1 kg and 171.3 cm, respectively, resulting in a median BMI of 24.2 kg/m^2^ (IQR = 5.1). Most participants had normal weight (64.0%), while 26.7% were overweight, 7.5% were obese, and 1.8% were underweight. With regard to negative lifestyle habits, 25.4% of participants reported cigarette smoking, 15.3% reported e-cigarette use, and 8.2% reported alcohol consumption. Self-rated health was most frequently assessed as very good (54.6%) or excellent (27.5%) (Table 1).

Differences between women and men were observed across several lifestyle and anthropometric characteristics. Women had a slightly lower median attitude toward a healthy lifestyle compared with men (Mdn = 51.0, IQR = 9.0, Mean Rank = 278.8 for women vs. Mdn = 53.0, IQR = 8.5, Mean Rank = 320.3 for men; *p* = 0.027). Women also reported shorter sleep duration than men (Mdn = 7.0, IQR = 2.0, Mean Rank = 275.5 for women vs. Mdn = 7.0, IQR = 1.0, Mean Rank = 319.4 for men; *p* = 0.014) (Table 1).

Men were significantly taller than women (Mdn = 1.84, IQR = 0.10, Mean Rank = 493.7 for men vs. Mdn = 1.69, IQR = 0.37, Mean Rank = 245.4 for women; *p* < 0.001) and had higher body weight (Mdn = 85.0, IQR = 14.5, Mean Rank = 469.2 for men vs. Mdn = 67.0, IQR = 15.0, Mean Rank = 250.1 for women; *p* < 0.001). Accordingly, men also had a higher BMI compared with women (Mdn = 25.3, IQR = 5.2, Mean Rank = 376.3 for men vs. Mdn = 23.1, IQR = 4.5, Mean Rank = 268.0 for women; *p* < 0.001). Men reported a higher prevalence of overweight (41.3% vs. 23.8%) and obesity (12.0% vs. 6.7%) than women (*p* < 0.001 for both). Alcohol consumption was also more frequent among men (13.0% vs. 7.3%; *p* = 0.030). In addition, men more often rated their health as excellent (51.1% vs. 23.0%; *p* < 0.001), whereas women more frequently reported very good self-rated health (57.0% vs. 40.2%; *p* < 0.001) (Table 1).

### 3.3. Differences in Attitude Toward a Healthy Lifestyle and Lifestyle Behaviors According to Sociodemographic and Lifestyle Characteristics

Age-related differences were observed across several lifestyle characteristics. The lowest daily meal frequency was observed among participants aged 46–60 years (Mdn = 3.0, IQR = 1.0, Mean Rank = 247.1), whereas a higher frequency was reported by participants aged 20 years or younger (Mdn = 3.0, IQR = 1.0, Mean Rank = 329.6), *p* = 0.002. Participants aged 61–75 years reported shorter sleep duration (Mdn = 7.0, IQR = 2.0, Mean Rank = 237.7, *p* = 0.011) and longer sitting time (Mdn = 8.0, IQR = 5.0, Mean Rank = 343.2, *p* = 0.004) compared with other age groups, and also had a significantly higher BMI (Mdn = 25.3, IQR = 6.5, Mean Rank = 361.5, *p* < 0.001) (Table 2).

Participants living in urban areas reported significantly longer sitting time than those living in rural areas (Mdn = 6.0, IQR = 4.0, Mean Rank = 291.0 vs. Mdn = 5.0, IQR = 3.0, Mean Rank = 231.6; *p* < 0.001). Individuals with a university education also reported longer sitting time (Mdn = 6.0, IQR = 4.0, Mean Rank = 297.2, *p* < 0.001) and a more favorable attitude toward a healthy lifestyle (Mdn = 52.0, IQR = 10.0, Mean Rank = 306.0, *p* < 0.001). Participants employed in sedentary occupations reported longer sitting time than those in more physically active jobs (Mdn = 8.0, IQR = 4.0, Mean Rank = 359.8 vs. Mdn = 5.0, IQR = 3.0, Mean Rank = 212.9; *p* < 0.001).

Retired individuals reported shorter sleep duration (Mdn = 6.5, IQR = 4.0, Mean Rank = 226.0) and higher BMI (Mdn = 26.4, IQR = 5.8, Mean Rank = 352.8) compared with unemployed participants (*p* < 0.001, for both). Participants caring for a child with a chronic disease had lower BMI (Mdn = 22.9, IQR = 6.0, Mean Rank = 213.4, *p* = 0.036) and shorter sleep duration (Mdn = 7.0, IQR = 2.0, Mean Rank = 205.8, *p* = 0.022). Caregivers of ill adult family members and older persons also reported shorter sleep duration and less favorable attitude toward a healthy lifestyle (Table 2).

Smokers had a significantly lower daily meal frequency and less favorable attitude toward a healthy lifestyle (*p* < 0.001 for both). Participants who did not smoke reported a higher number of daily meals (Mdn = 3.0, IQR = 1.0, Mean Rank = 302.3) compared with regular smokers (Mdn = 3.0, IQR = 1.0, Mean Rank = 237.5, *p* < 0.001). Non-smokers also reported more favorable attitudes toward a healthy lifestyle (Mdn = 53.0, IQR = 10.0, Mean Rank = 320.6) than regular smokers (Mdn = 49.0, IQR = 8.0, Mean Rank = 213.5, *p* < 0.001) (Table 3).

E-cigarette users reported fewer daily meals (Mdn = 3.0, IQR = 1.0, Mean Rank = 256.7) compared with non-users (Mdn = 3.0, IQR = 1.0, Mean Rank = 295.4, *p* = 0.009). Additionally, e-cigarette users reported less favorable attitude toward a healthy lifestyle (Mdn = 49.0, IQR = 9.5, Mean Rank = 231.8, *p* = 0.004) and had a lower BMI (Mdn = 22.5, IQR = 3.5, Mean Rank = 238.9, *p* = 0.006).

No significant associations were observed between alcohol consumption and attitude toward a healthy lifestyle, number of daily meals, sitting time, sleep duration, or BMI.

Participants with better self-rated health reported significantly longer sleep duration (*p* < 0.001). Those who rated their health as excellent reported the longest sleep duration (Mdn = 7.0, IQR = 1.0, Mean Rank = 335.1) compared with participants reporting poor (Mdn = 6.0, IQR = 1.0, Mean Rank = 178.8) or average health (Mdn = 7.0, IQR = 1.0, Mean Rank = 225.7); *p* < 0.001. Participants with excellent self-rated health also exhibited more favorable attitude toward a healthy lifestyle (Mdn = 54.0, IQR = 11.5, Mean Rank = 348.8) than those with poor self-rated health (Mdn = 47.0, IQR = 11.5, Mean Rank = 170.0); *p* < 0.001.

Finally, higher BMI was associated with less favorable attitude toward a healthy lifestyle (*p* < 0.001). Participants with normal body weight (BMI 18.5–24.9 kg/m^2^) reported the most favorable attitude toward a healthy lifestyle, whereas individuals with obesity (BMI ≥ 30.0 kg/m^2^) demonstrated the least favorable attitude (Mdn = 53.0, IQR = 10.0, Mean Rank = 307.3 vs. Mdn = 49.0, IQR = 6.0, Mean Rank = 213.1; *p* < 0.001) (Table 3).

### 3.4. Associations Between Attitude Toward a Healthy Lifestyle, Lifestyle Behaviors, and BMI

After adjusting for age, sex, employment status, educational level, and type of work, a more positive attitude toward a healthy lifestyle was significantly associated with a higher number of daily meals (R = 0.20, *p* < 0.001), longer sleep duration (R = 0.25, *p* < 0.001), shorter sitting time (R = −0.12, *p* = 0.007), and lower BMI (R = −0.22, *p* < 0.001). In addition, a higher number of daily meals was significantly associated with longer sleep duration (R = 0.10, *p* = 0.016) (Supplementary Table S3).

Linear regression analyses showed that age was positively associated with attitude toward a healthy lifestyle (β = 0.21, *p* < 0.001) and BMI (β = 0.27, *p* < 0.001) and negatively associated with the number of daily meals (β = −0.26, *p* < 0.001). Sex was a significant predictor only for BMI, with female participants having a lower BMI compared with male participants (β = −0.24, *p* < 0.001). Living in an urban area was associated with longer sitting time (β = 0.10, *p* = 0.008), while being married or in a relationship was associated with longer sleep duration (β = 0.11, *p* = 0.011). Being employed was associated with shorter sitting time (β = −0.17, *p* < 0.001), shorter sleep duration (β = −0.10, *p* = 0.045), and higher BMI (β = 0.14, *p* = 0.003). Sedentary work was associated with longer sitting time (β = 0.43, *p* < 0.001). Caring for a child with a chronic disease was associated with a lower BMI (β = −0.13, *p* = 0.001) (Table 4).

Regarding negative lifestyle behaviors, cigarette smoking was negatively associated with attitude toward a healthy lifestyle (β = −0.18, *p* < 0.001), whereas e-cigarette use was negatively associated with the number of daily meals (β = −0.10, *p* = 0.025). Alcohol consumption was negatively associated with BMI (β = −0.08, *p* = 0.044). Among lifestyle variables, attitude toward a healthy lifestyle was a significant predictor of the number of daily meals (β = 0.16, *p* = 0.001), sitting time (β = −0.11, *p* = 0.010), sleep duration (β = 0.17, *p* < 0.001), and BMI (β = −0.24, *p* < 0.001). Excellent self-rated health was positively associated with attitude toward a healthy lifestyle (β = 0.19, *p* < 0.001) and sleep duration (β = 0.09, *p* = 0.038) (Table 4).

### 3.5. Associations Between Healthy Lifestyle Behaviors, Excess Body Weight, and Self-Rated Health

Increasing age was significantly associated with excess body weight (OR = 1.06; 95% CI = 1.03–1.08; *p* < 0.001), indicating that the likelihood of excess body weight increased by approximately 6% with each additional year of age. Sex was also a significant factor, with female participants being significantly less likely to have excess body weight compared with male participants (OR = 0.23; 95% CI = 0.13–0.41; *p* < 0.001). Being employed was associated with a higher likelihood of excess body weight (OR = 2.39; 95% CI = 1.20–4.75; *p* = 0.013). Current e-cigarette use was associated with a lower likelihood of excess body weight (OR = 0.53; 95% CI = 0.30–0.93; *p* = 0.028). Attitude toward a healthy lifestyle was also a significant predictor, with a more positive attitude associated with a lower likelihood of excess body weight (OR = 0.92; 95% CI = 0.89–0.95; *p* < 0.001). No other examined characteristics were significantly associated with excess body weight (Table 5).

With regard to self-rated health, female participants had significantly lower odds of reporting excellent self-rated health compared with male participants (OR = 0.25; *p* < 0.001), indicating a 75% reduction in the odds of excellent self-rated health (Table 6). Age was a significant negative predictor of excellent self-rated health (OR = 0.94; *p* < 0.001), with each additional year of age associated with an approximate 6% decrease in the odds of reporting excellent health. Longer daily sleep duration was associated with higher odds of excellent self-rated health (OR = 1.31; *p* = 0.014), with each additional hour of sleep increasing the odds by approximately 31%. Higher BMI was also associated with greater odds of reporting excellent self-rated health (OR = 1.08; *p* < 0.001), corresponding to an 8% increase in odds per unit increase in BMI. No other variables were significantly associated with excellent self-rated health (Table 6).

## 4. Discussion

This study examined the relationships between attitude toward a healthy lifestyle, lifestyle behaviors, sociodemographic characteristics, and BMI in a community-based sample of adults. The findings indicate that a more positive attitude toward a healthy lifestyle is associated with healthier behavioral patterns and more favorable weight status. Participants with a more positive attitude reported a higher number of daily meals, longer sleep duration, shorter sitting time, and lower BMI. Overall, attitude toward a healthy lifestyle emerged as an important psychological factor linked to everyday lifestyle behaviors and body weight.

**Figure 1:**
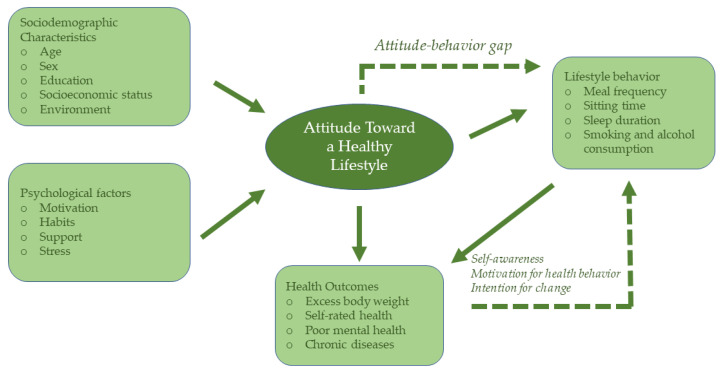
Conceptual model of the associations between attitude toward a healthy lifestyle, lifestyle behaviors, and health outcomes.

Participants demonstrated a moderate to slightly above-average positive attitude toward a healthy lifestyle, suggesting awareness of the importance of healthy behaviors without full translation into optimal lifestyle practices. Although positive attitudes were associated with healthier behaviors in this study, previous research has documented an attitude-behavior gap in lifestyle research, whereby positive attitudes do not necessarily lead to sustained health-promoting behaviors due to barriers such as limited time, occupational demands, lack of motivation, and social or cultural constraints [53,54,55]. From a behavioral theory perspective, these findings indicate that attitude toward a healthy lifestyle represents an important but not sufficient determinant of behavior, functioning as a mediating psychological factor that shapes intentions while contextual and structural barriers influence whether attitudes are translated into sustained lifestyle change. Figure 1 provides a conceptual overview of the hypothesized relationships between attitude toward a healthy lifestyle, lifestyle behaviors, sociodemographic factors, and health outcomes, highlighting the attitude–behavior gap. The observed association between a more positive attitude and lower odds of excess body weight underscores the relevance of behavioral awareness and motivation in shaping weight-related outcomes.

The results highlight the complex interplay between psychological, behavioral, and sociodemographic factors in shaping lifestyle patterns and body weight. In the present study, lifestyle behaviors were more strongly associated with attitude toward a healthy lifestyle than with BMI. This finding likely reflects the cumulative nature of BMI and its limited sensitivity to current behavioral patterns, whereas attitude toward a healthy lifestyle represents a more proximal determinant of daily health-related behaviors [43,56]. It is important to note that BMI reflects the cumulative effects of multiple dietary and nutritional factors, including diet quality, overall energy intake, and macronutrient balance, which were not directly assessed in the present study. Participants with normal body weight exhibited more favorable attitudes toward a healthy lifestyle, supporting previous evidence that health-oriented values are linked to more adaptive behavioral profiles [57].

Age was significantly associated with several lifestyle-related outcomes. With increasing age, participants exhibited higher BMI values, while the number of daily meals tended to be lowest among middle-aged participants (46–60 years). Age-related differences were also observed in sleep duration and sitting time. These findings are consistent with previous research showing that aging is associated with progressive changes in metabolism, body composition, appetite regulation, and physical activity patterns, which collectively contribute to altered lifestyle and health outcomes [58,59]. The observed association between older age and lower self-rated health further supports the cumulative impact of aging on metabolic health and overall well-being [59].

Sleep was identified as a particularly important factor for subjective health. Although sleep duration was not directly associated with BMI, longer sleep duration was strongly associated with excellent self-rated health. Adequate sleep supports metabolic regulation, immune function, and mental health [60], and age-related changes in circadian rhythms, sleep fragmentation, and sedentary behavior may contribute to shorter sleep duration in older adults [61]. These findings underscore the central role of sleep in perceived health and overall well-being.

In this study, meal frequency, sitting time, and sleep duration were selected as simple and commonly used behavioral markers of dietary habits and everyday lifestyle patterns, which have been linked in previous research to metabolic health, body weight regulation, and health-related behaviors [62,63,64]. The observed positive association between a higher number of daily meals and longer sleep duration may reflect more structured daily routines, whereby individuals who consume meals more regularly also maintain healthier behavioral patterns and circadian rhythms that support adequate sleep. In addition, meal timing and composition may influence satiety, metabolic regulation, and glycemic stability, which could indirectly affect sleep duration [62,63,64,65,66,67]. Given the cross-sectional design, these findings should be interpreted as exploratory and hypothesis-generating.

Sedentary behavior was also closely linked to attitude toward a healthy lifestyle. Participants with a more positive attitude reported shorter sitting time, suggesting that health-oriented beliefs may be linked to reduced sedentary behavior [36,68]. This association highlights the potential role of attitudes toward a healthy lifestyle in shaping everyday movement patterns beyond structured physical activity and underscores the importance of targeting sedentary behavior as a distinct component of lifestyle interventions [69,70]. Employment status and type of work further influenced sedentary behavior, with sedentary occupations associated with longer sitting time. Sedentary behavior is a multidimensional pattern occurring across occupational and non-occupational contexts, and individuals with sedentary jobs may accumulate additional sitting time outside work, increasing overall physical inactivity. Accordingly, sedentary work and total sitting time were included simultaneously in the regression model to distinguish occupational sitting from overall sedentary exposure and to assess their independent contributions, despite expected overlap. This interpretation aligns with previous evidence showing higher total sitting time and lower physical activity among individuals in sedentary occupations, particularly office workers [71,72,73].

Sex differences were evident across several outcomes. Male participants exhibited higher BMI, longer sleep duration, and higher odds of reporting excellent self-rated health, while female participants were less likely to have excess body weight. These findings suggest discrepancies between objective and subjective health indicators. Although BMI is widely used in population health research, it does not account for body composition, cardiorespiratory fitness, or fat distribution [47,74]. Individuals with higher BMI can still exhibit favorable health profiles when higher body weight reflects a favorable distribution of muscle and fat or adequate fitness levels [75,76]. In this context, differences in body composition, particularly higher muscle mass among men, may partially explain elevated BMI values without necessarily indicating excess adiposity [77,78]. In addition, men may perceive their health more positively despite higher BMI, whereas sociocultural pressures and hormonal factors may influence health perceptions and weight-related behaviors among women [79,80]. These findings highlight the importance of considering both objective and subjective health indicators in public health strategies.

Urban residence and higher education were associated with longer sitting time, likely reflecting occupational and lifestyle characteristics common in urban and professional settings. While no association was found between residential environment and meal frequency in the present study, previous research reports mixed findings regarding urban-rural differences in dietary patterns [81,82]. For example, a descriptive analytical study comparing the number of daily meals confirmed that people in rural areas tend to have a higher number of daily meals than those in urban areas [82]. A study conducted in Croatia indicated that participants living in urban areas consume more fruits and vegetables compared to those from rural areas, suggesting differences in dietary habits and diet quality between these populations [83].

Caregiving responsibilities also influenced lifestyle outcomes. Caring for a child with a chronic disease was associated with lower BMI, potentially reflecting caregiving burden, increased stress, and disruptions in daily routines [33,35]. These findings emphasize the need to consider caregiving roles as important social-contextual factors when addressing lifestyle behaviors and health risks.

Unhealthy lifestyle habits further shaped attitudes and behaviors. Smokers reported a less positive attitude toward a healthy lifestyle, consistent with the well-documented clustering of smoking with other adverse lifestyle behaviors. E-cigarette use was associated with fewer daily meals and lower BMI, potentially reflecting nicotine-related appetite suppression and irregular eating patterns [84,85]. Notably, the previously reported lower BMI among e-cigarette users supports the association observed in the present study. These findings should be interpreted cautiously, as lower BMI in this context does not necessarily indicate better health.

Subjective health perception appeared to integrate multiple dimensions of lifestyle and health. While higher BMI was associated with less favorable attitude toward a healthy lifestyle, it was also linked to higher odds of reporting excellent self-rated health. This apparent paradox likely reflects limitations of BMI as a sole indicator of health, as BMI does not differentiate between fat and lean mass and may misclassify individuals with higher muscle mass [86]. Discrepancies between anthropometric measures and perceived health underscore the importance of using multidimensional health indicators in research and practice.

The findings of this study have important implications for lifestyle medicine and public health. Lifestyle medicine emphasizes behavior modification through multidisciplinary approaches aimed at preventing and managing chronic diseases [87]. While healthy habits substantially reduce chronic disease risk, sustained behavior change remains challenging, particularly when negative habits coexist [19]. The Health Belief Model suggests that behavior change occurs when individuals perceive their current habits as a health threat and believe that change will yield meaningful benefits [57]. Integrating a salutogenic approach further emphasizes the identification of positive resources that enable individuals to maintain and strengthen their health.

The present study has several limitations that should be acknowledged. The cross-sectional design prevents conclusions about causality, and the observed associations should therefore be interpreted as exploratory. The use of a non-random, convenience online sample may have introduced selection bias, potentially leading to imbalances across sex, age, education, and other sociodemographic characteristics, as well as overrepresentation of health-conscious individuals. Consequently, the sample may not be fully representative of the general population, which may limit the generalizability of the findings. Reliance on self-reported data may have resulted in recall or social desirability bias, particularly for sensitive information such as body weight and lifestyle behaviors. Dietary intake was not comprehensively assessed. Dietary behavior was assessed solely by meal frequency, without information on diet intake, diet quality, meal composition, timing, or energy intake, which limits the interpretative value of findings related to eating behavior. Similarly, sleep duration and sedentary behavior were assessed using self-reported measures, and other relevant contextual factors, such as sleep quality, physical activity intensity, or occupational demands, were not captured and may have influenced the observed associations. In addition, personality traits were not assessed, although attitude toward a healthy lifestyle may depend on individual psychological characteristics. Some relevant confounders, including genetic predisposition, detailed body composition measures, and psychosocial variables, were also not included in the analyses. Future longitudinal studies incorporating objective and more comprehensive measures of lifestyle behaviors and health indicators are needed to confirm these findings and to further explore causal pathways.

Despite these limitations, this study has notable strengths, including the simultaneous examination of multiple lifestyle dimensions and robust statistical analyses. By highlighting the role of attitude toward a healthy lifestyle in shaping behaviors, weight status, and perceived health, these findings provide insights for the development of targeted interventions and public health strategies aimed at promoting sustainable lifestyle change.

To enhance the public health relevance of these findings, it is important to consider specific intervention strategies targeting lifestyle behaviors, including sedentary time, physical activity, and sleep. Behavior change interventions incorporating personalized feedback, education, and counselling can help reduce sedentary behavior and promote more active lifestyles, while education on sleep hygiene and sleep health may improve sleep quality and duration [88,89,90]. Additionally, health promotion campaigns and multi-component lifestyle management programmes addressing multiple behaviors simultaneously, such as physical activity, nutrition, sedentary behavior, and sleep, have the potential to shift attitudes toward healthier lifestyles and support sustained behavior change at the population level [88,89,90]. Furthermore, education campaigns targeting lifestyle attitudes should be implemented across the lifespan, beginning in early childhood and school-age populations [91,92] and continuing through adulthood and older age to ensure that healthy behaviors are promoted and reinforced at all stages of life [93].

## 5. Conclusions

The present study demonstrated that attitude toward a healthy lifestyle was significantly associated with lifestyle behaviors, BMI, excess body weight, and self-rated health in a community-based adult sample. A more positive attitude toward a healthy lifestyle was associated with a higher number of daily meals, longer sleep duration, shorter sitting time, lower BMI, and lower odds of excess body weight. In addition, self-rated health was positively associated with longer sleep duration and a more favorable attitude toward a healthy lifestyle. Lifestyle behaviors, body weight indicators, and perceived health varied across sociodemographic and social role-related factors, including age, sex, employment status, type of work, place of residence, caregiving responsibilities, and health-related habits such as smoking and e-cigarette use. This study contributes to the existing literature by examining attitude toward a healthy lifestyle alongside multiple everyday lifestyle behaviors simultaneously in a Croatian adult population, providing insights that can inform targeted public health interventions. These findings highlight attitude toward a healthy lifestyle as an important psychological correlate of everyday behaviors and perceived health; however, given the cross-sectional design, the observed associations should be interpreted as non-causal.

From a public health and lifestyle medicine perspective, the results underscore the need for targeted, actionable strategies that go beyond increasing awareness alone. Interventions may include workplace-based programs aimed at reducing sitting time, initiatives promoting healthy dietary habits, sleep hygiene and adequate sleep duration, and educational campaigns designed to foster positive lifestyle attitudes while addressing occupational, social, and caregiving-related barriers to behavior change. Future longitudinal and interventional studies incorporating objective measures of lifestyle behaviors and body composition are needed to clarify causal relationships and to support the development of effective strategies for promoting sustainable healthy lifestyles and improving population health.

## Figures and Tables

**Table 1:** Attitude toward a healthy lifestyle and lifestyle characteristics in the total sample and among women and men (N = 570).

	Total Sample (N = 570)	Women (N = 478)	Men (N = 92)	* p *
Attitude toward a healthy lifestyle (score *), Mdn (IQR); Mean Rank	52.0 (10.0)	51.0 (9.0) 278.8	53.0 (8.5) 320.3	0.027 ^†^
Meal frequency (number of daily meals), Mdn (IQR); Mean Rank	3.0 (1.0)	3.0 (1.0) 284.6	3.0 (1.0) 290.4	0.738 ^†^
Sitting time (hours per day), Mdn (IQR); Mean Rank	5.0 (4.0)	5.0 (4.0) 277.0	5.0 (4.0) 274.1	0.879 ^†^
Sleep duration (hours per day), Mdn (IQR); Mean Rank	7.0 (2.0)	7.0 (2.0) 275.5	7.0 (1.0) 319.4	0.014 ^†^
Body height (m), Mdn (IQR); Mean Rank	1.71 (0.10)	1.69 (0.37) 245.4	1.84 (0.10) 493.7	<0.001 ^†^
Body weight (kg), Mdn (IQR); Mean Rank	73.1 (17.0)	67.0 (15.0) 250.1	85.0 (14.5) 469.2	<0.001 ^†^
BMI (kg/m^2^), Mdn (IQR); Mean Rank	24.2 (5.1)	23.1 (4.5) 268.0	25.3 (5.2) 376.3	<0.001 ^†^
BMI category, N (%)				
Underweight (<18.5 kg/m^2^)	10 (1.8)	10 (2.1)	0 (0.0)	
Normal weight (18.5–24.9 kg/m^2^)	365 (64.0)	322 (67.4)	43 (46.7)	<0.001 ^‡^
Overweight (25.0–29.9 kg/m^2^)	152 (26.7)	114 (23.8)	38 (41.3)	
Obesity (≥30.0 kg/m^2^)	43 (7.5)	32 (6.7)	11 (12.0)	
Cigarette smoking, N (%)				
No	365 (64.0)	303 (63.4)	62 (67.4)	
Yes	145 (25.4)	126 (26.4)	19 (20.7)	0.498 ^‡^
Occasionally	60 (10.5)	49 (10.3)	11 (12.0)	
E-cigarette use, N (%)				
No	450 (78.9)	371 (77.6)	79 (85.9)	
Yes	87 (15.3)	79 (16.5)	8 (8.7)	0.150 ^‡^
Occasionally	33 (5.8)	28 (5.9)	5 (5.4)	
Alcohol consumption, N (%)				
No	188 (33.0)	167 (34.9)	21 (22.8)	
Yes	47 (8.2)	35 (7.3)	12 (13.0)	0.030 ^‡^
Occasionally	335 (58.8)	276 (57.7)	59 (64.1)	
Self-rated health, N (%)				
Excellent	157 (27.5)	110 (23.0)	47 (51.1)	
Very good	311 (54.6)	274 (57.3)	37 (40.2)	<0.001 ^‡^
Average	92 (16.1)	86 (18.0)	6 (6.5)	
Poor	10 (1.8)	8 (1.7)	2 (2.2)	

Note: N = number of participants; Mdn = median; IQR = interquartile range; Mean Rank = average rank (women vs. men only); BMI = Body Mass Index; * possible score range: 15–75; ^†^ Mann-Whitney U test, ^‡^ Chi-square test.

**Table 2:** Differences in attitude toward a healthy lifestyle, lifestyle behaviors, and body mass index according to sociodemographic characteristics and caregiving responsibilities (N = 570).

	Number of Daily Meals		Sitting Time		Sleep Duration		BMI		Attitude Toward a Healthy Lifestyle	
	Mdn (IQR); Mean Rank	*p* Value	Mdn (IQR); Mean Rank	*p* Value	Mdn (IQR); Mean Rank	*p* Value	Mdn (IQR); Mean Rank	*p* Value	Mdn (IQR); Mean Rank	*p* Value
Age group (years)										
≤20	3.0 (1.0); 329.6	0.002	6.0 (3.0); 308.6	0.004	8.0 (1.0); 329.4	0.011	22.0 (3.2); 193.5	<0.001	50.0 (8.0); 223.1	0.095
21–30	3.0 (1.0); 302.1		5.0 (4.0); 231.1		7.0 (1.0); 313.2		22.4 (4.1); 226.4		52.0 (10.5); 278.2	
31–45	3.0 (1.0); 297.8		6.0 (4.0); 282.8		7.0 (2.0); 277.3		23.5 (4.5); 287.9		52.0 (9.6); 300.4	
46–60	3.0 (0.8); 247.1		5.0 (4.0); 282.8		7.0 (2.0); 261.9		24.8 (5.3); 338.5		51.0 (9.5); 285.9	
61–75	3.0 (1.8); 261.3		8.0 (5.0); 343.2		7.0 (2.0); 237.7		25.3 (6.5); 361.5		51.0 (9.5); 279.0	
Place of residence										
Rural	3.0 (1.0); 268.8	0.139	5.0 (3.0); 231.6	<0.001	7.0 (2.0); 286.3	0.741	24.2 (4.5); 303.1	0.145	50.0 (8.0); 262.4	0.056
Urban	3.0 (1.0); 290.9		6.0 (4.0); 291.0		7.0 (2.0); 281.3		23.4 (4.8); 279.8		52.0 (10.0); 293.0	
Marital status										
Divorced, widowed, single	3.0 (1.0); 288.3	0.800	5.0 (4.0); 274.6	0.869	7.0 (2.0); 273.5	0.428	23.2 (4.6); 271.1	0.226	50.0 (10.0); 263.4	0.064
Married/in a relationship	3.0 (1.0); 284.6		5.0 (4.0); 277.1		7.0 (2.0); 285.5		23.8 (4.9); 290.3		52.0 (9.0); 292.9	
Education level										
Primary and high school	3.0 (1.0); 269.5	0.093	5.0 (3.0); 229.7	<0.001	7.0 (2.0); 283.6	0.907	24.5 (4.8); 278.4	0.485	50.0 (8.0); 240.7	<0.001
University	3.0 (1.0); 292.8		6.0 (4.0); 297.2		7.0 (2.0); 282.0		24.8 (5.3); 288.8		52.0 (10.0); 306.0	
Employment status										
Unemployed	3.0 (1.0); 271.7	0.454	5.0 (4.0); 249.5	0.374	7.0 (1.0); 337.2	<0.001	21.6 (3.9); 191.6	<0.001	51.5 (7.5); 296.9	0.804
Employed	3.0 (1.0); 282.6		5.0 (4.0); 276.9		7.0 (1.0); 271.5		23.4 (4.8); 301.8		52.0 (10.0); 287.0	
Student	3.0 (1.0); 315.8		6.0 (2.0); 299.9		8.0 (1.0); 360.4		22.0 (3.5); 183.5		50.0 (10.3); 265.5	
Retired	3.0 (1.0); 299.1		5.0 (6.0); 225.8		6.5 (4.0); 226.0		26.4 (5.8); 352.8		51.5 (12.3); 285.6	
Type of work										
Active	3.0 (1.0); 291.4	0.285	5.0 (3.0); 212.9	<0.001	7.0 (2.0); 282.2	0.961	23.2 (4.6); 273.0	0.036	51.0 (9.0); 281.1	0.459
Sedentary	3.0 (1.0); 277.6		8.0 (4.0); 359.8		7.0 (2.0); 282.9		24.0 (4.9); 302.2		52.0 (9.6); 291.4	
Caring for a child with a disability										
No	3.0 (1.0); 286.4	0.505	5.0 (4.0); 276.4	0.947	7.0 (2.0); 284.2	0.208	23.7 (4.8); 287.8	0.103	52.0 (10.0); 286.2	0.626
Yes	3.0 (1.0); 264.6		5.0 (4.0); 278.7		7.0 (2.0); 241.5		22.6 (5.7); 230.7		52.0 (9.0); 269.1	
Caring for a child with a chronic disease										
No	3.0 (1.0); 287.5	0.118	5.0 (4.0); 276.2	0.820	7.0 (2.0); 285.5	0.022	23.6 (4.9); 288.4	0.036	52.0 (10.0); 287.1	0.260
Yes	3.0 (0.8); 235.4		5.0 (5.0); 284.2		7.0 (2.0); 205.8		22.9 (6.0); 213.4		49.0 (10.5); 246.7	
Caring for an ill adult family member										
No	3.0 (1.0); 287.2	0.416	5.0 (4.0); 277.7	0.589	7.0 (2.0); 289.8	0.001	23.5 (4.6); 284.3	0.593	52.0 (9.0); 292.8	<0.001
Yes	3.0 (2.0); 269.3		5.0 (4.0); 265.3		7.0 (1.0); 212.4		24.6 (7.1); 296.9		48.0 (8.8); 216.3	
Caring for an older person										
No	3.0 (1.0); 288.9	0.159	5.0 (4.0); 281.9	0.033	7.0 (2.0); 289.8	0.003	23.6 (4.7); 285.6	0.926	52.0 (9.0); 292.1	0.010
Yes	3.0 (1.0); 261.1		5.0 (5.0); 238.5		7.0 (2.0); 228.9		24.0 (6.0); 287.2		50.0 (8.0); 237.8	

Note: Mdn = median; IQR = interquartile range; BMI = Body Mass Index.

**Table 3:** Differences in attitude toward a healthy lifestyle, lifestyle behaviors, and body mass index according to lifestyle characteristics (N = 570).

	Number of Daily Meals		Sitting Time		Sleep Duration		BMI		Attitude Toward a Healthy Lifestyle	
	Mdn (IQR); Mean Rank	*p* Value	Mdn (IQR); Mean Rank	*p* Value	Mdn (IQR); Mean Rank	*p* Value	Mdn (IQR); Mean Rank	*p* Value	Mdn (IQR); Mean Rank	*p* Value
Cigarette smoking										
No	3.0 (1.0); 302.3		5.0 (4.0); 277.1		7.0 (2.0); 289.6		23.6 (4.6); 284.1		53.0 (10.0); 320.6	
Yes	3.0 (1.0); 237.5	<0.001	5.0 (4.0); 262.5	0.196	7.0 (2.0); 268.5	0.348	24.0 (6.1); 298.6	0.346	49.0 (8.0); 213.5	<0.001
Occasionally	3.0 (1.0); 299.2		5.0 (4.0); 307.2		7.0 (2.0); 273.3		22.9 (4.4); 262.4		49.0 (7.0); 245.8	
E-cigarette use										
No	3.0 (1.0); 295.4	0.009	5.0 (4.0); 272.0	0.252	7.0 (2.0); 284.4	0.491	24.0 (4.9); 296.8	0.006	52.0 (9.0); 296.3	0.004
Yes	3.0 (1.5); 253.7		6.0 (4.0); 284.4		7.0 (2.0); 266.0		22.5 (3.5); 238.9		49.0 (9.5); 231.8	
Occasionally	3.0 (1.0); 233.9		6.0 (5.0); 318.0		7.0 (1.0); 299.8		22.7 (3.4); 255.1		51.0 (11.0); 279.4	
Alcohol consumption										
No	3.0 (1.0); 300.3	0.054	5.0 (5.0); 257.6	0.051	7.0 (2.0); 275.1	0.366	24.2 (4.5); 302.0	0.156	52.0 (9.0); 300.5	0.147
Yes	3.0 (1.0); 240.7		6.0 (3.0); 318.0		7.0 (1.0); 311.1		24.0 (4.5); 298.8		51.0 (7.5); 250.6	
Occasionally	3.0 (1.0); 283.5		5.0 (4.0); 281.3		7.0 (2.0); 282.7		23.1 (4.3); 274.4		51.0 (10.0); 282.0	
BMI category										
<18.5 kg/m^2^	3.0 (1.0); 286.5	0.926	6.0 (6.0); 349.1	0.328	8.0 (3.0); 290.3	0.597	18.3 (4.5); 5.50	<0.001	54.0 (9.5); 259.4	<0.001
18.5–24.9 kg/m^2^	3.0 (1.0); 286.4		5.0 (4.0); 271.7		7.0 (2.0); 288.9		22.2 (2.5); 193.0		53.0 (10.0); 307.3	
25.0–29.9 kg/m^2^	3.0 (1.0); 287.6		5.0 (4.0); 275.9		7.0 (2.0); 271.7		27.1 (2.5); 451.5		50.0 (9.0); 255.4	
≥30.0 kg/m^2^	3.0 (1.0); 270.2		5.0 (5.0); 303.8		7.0 (2.0); 265.0		31.3 (3.6); 549.0		49.0 (6.0); 213.1	
Self-rated health										
Poor	3.0 (1.5); 202.0	0.066	5.0 (7.0); 224.6	0.609	6.0 (4.0); 178.8	<0.001	24.7 (8.3); 299.1	0.145	47.0 (11.5); 170.0	<0.001
Average	3.0 (1.3); 258.9		5.0 (4.0); 266.5		7.0 (1.0); 225.7		23.9 (6.0); 301.0		50.0 (8.5); 228.5	
Very good	3.0 (1.0); 288.9		6.0 (5.0); 282.5		7.0 (2.0); 275.8		23.9 (5.3); 293.4		51.0 (9.0); 274.2	
Excellent	3.0 (1.0); 299.6		5.0 (4.0); 273.1		7.0 (1.0); 335.1		23.0 (4.2); 259.9		54.0 (11.5); 348.8	

Note: Mdn = median; IQR = interquartile range; BMI = Body Mass Index.

**Table 4:** Associations between attitude toward a healthy lifestyle, lifestyle behaviors, and BMI based on linear regression models (N = 570).

	Attitude Toward a Healthy Lifestyle		Number of Daily Meals		Sitting Time		Sleep Duration		BMI	
	β	*p*	β	*p*	β	*p*	β	*p*	β	*p*
Age	0.21	<0.001	−0.26	<0.001	0.10	0.057	−0.07	0.217	0.27	<0.001
Female (male as reference)	−0.03	0.513	−0.01	0.881	0.07	0.084	−0.04	0.339	−0.24	<0.001
Urban (rural as reference)	0.01	0.755	0.07	0.107	0.10	0.008	−0.04	0.395	−0.06	0.156
Married/in a relationship (no as reference)	0.02	0.556	−0.07	0.140	0.02	0.708	0.11	0.011	−0.03	0.545
Having children (yes)	−0.03	0.512	0.09	0.114	−0.07	0.163	−0.17	0.002	0.02	0.734
University degree (no university as reference)	0.11	0.013	0.07	0.143	0.08	0.068	0.03	0.556	−0.01	0.786
Employed (unemployed as reference)	−0.03	0.495	0.00	0.984	−0.17	<0.001	−0.10	0.045	0.14	0.003
Sedentary work (yes)	0.01	0.881	−0.06	0.270	0.43	<0.001	0.03	0.569	−0.05	0.256
Caring for a child with a disability (yes)	0.00	0.926	−0.01	0.822	0.00	0.939	−0.01	0.781	0.03	0.489
Caring for a child with a chronic disease (yes)	−0.04	0.345	−0.04	0.443	0.01	0.776	−0.06	0.155	−0.13	0.001
Caring for an ill adult family member (yes)	−0.07	0.099	0.04	0.422	0.00	0.987	−0.09	0.073	0.00	0.933
Caring for an older person (yes)	−0.02	0.668	−0.03	0.535	−0.08	0.073	−0.04	0.391	−0.02	0.705
Cigarette smoking (yes)	−0.18	<0.001	−0.07	0.097	−0.01	0.859	0.00	0.990	−0.01	0.872
E-cigarette use (yes)	−0.03	0.453	−0.10	0.025	0.05	0.190	−0.03	0.444	−0.02	0.650
Alcohol consumption (yes)	−0.02	0.599	−0.08	0.071	0.05	0.180	0.02	0.564	−0.08	0.044
Excellent self-rated health (yes)	0.19	<0.001	−0.08	0.103	0.02	0.566	0.09	0.038	−0.06	0.165
Attitude toward a healthy lifestyle (score)	-	-	0.16	0.001	−0.11	0.010	0.17	<0.001	−0.24	<0.001
Number of daily meals	0.13	0.001	-	-	0.00	0.905	0.05	0.249	0.03	0.499
Sitting time (hours per day)	−0.11	0.010	0.01	0.905	-	-	0.08	0.074	0.04	0.334
Sleep duration (hours per day)	0.15	<0.001	0.05	0.249	0.07	0.074	-	-	0.02	0.662
BMI	−0.23	<0.001	0.03	0.499	0.04	0.334	0.02	0.662	-	-

Note: BMI = Body Mass Index; β = Beta coefficient; *p* = *p* value.

**Table 5:** Logistic regression analysis of factors associated with excess body weight (N = 570).

	β	S.E.	Wald	OR (95% CI)	*p*
Age	0.05	0.01	22.08	1.06 (1.03–1.08)	<0.001
Female (male as reference)	−1.47	0.29	25.74	0.23 (0.13–0.41)	<0.001
Urban (rural as reference)	−0.37	0.24	2.23	0.69 (0.43–1.12)	0.135
Married/in a relationship (no as reference)	−0.10	0.26	0.14	0.91 (0.54–1.52)	0.710
Having children (yes)	0.05	0.28	0.03	1.05 (0.61–1.83)	0.855
University degree (no university as reference)	−0.25	0.25	0.98	0.78 (0.48–1.28)	0.323
Employed (unemployed as reference)	0.87	0.35	6.11	2.39 (1.20–4.75)	0.013
Sedentary work (yes)	−0.32	0.24	1.66	0.73 (0.45–1.18)	0.197
Caring for a child with a disability (yes)	−0.37	0.61	0.36	0.69 (0.21–2.31)	0.550
Caring for a child with a chronic disease (yes)	−0.95	0.63	2.24	0.39 (0.11–1.34)	0.134
Caring for an ill adult family member (yes)	−0.04	0.40	0.01	0.96 (0.44–2.10)	0.918
Caring for an older person (yes)	−0.15	0.36	0.17	0.86 (0.43–1.75)	0.680
Cigarette smoking (yes)	0.10	0.23	0.21	1.11 (0.71–1.73)	0.647
E-cigarette use (yes)	−0.64	0.29	4.82	0.53 (0.30–0.93)	0.028
Alcohol consumption (yes)	−0.37	0.23	2.66	0.69 (0.44–1.08)	0.103
Attitude toward a healthy lifestyle (score)	−0.08	0.02	22.96	0.92 (0.89–0.95)	<0.001
Number of daily meals	0.15	0.10	2.20	1.17 (0.95–1.43)	0.138
Sitting time (hours per day)	0.04	0.04	1.27	1.05 (0.97–1.13)	0.261
Sleep duration (hours per day)	−0.04	0.09	0.24	0.96 (0.80–1.14)	0.626
Excellent self-rated health (yes)	−0.19	0.27	0.51	0.83 (0.49–1.39)	0.477

Note: β = beta coefficient; S.E. = standard error; Wald = Wald test; OR (95% CI) = Odds ratio with 95% confidence interval; *p* = *p* value.

**Table 6:** Logistic regression analysis of factors associated with excellent self-rated health (N = 570).

	β	S.E.	Wald	OR (95%CI)	*p*
Age	−0.06	0.01	16.84	0.94 (0.91–0.97)	<0.001
Female (male as reference)	−1.40	0.30	22.01	0.25 (0.14–0.44)	<0.001
Urban (rural as reference)	0.16	0.27	0.34	1.17 (0.69–1.97)	0.562
Married/in a relationship (no as reference)	0.12	0.28	0.17	1.12 (0.65–1.94)	0.677
Having children (yes)	−0.11	0.29	0.15	0.89 (0.51–1.58)	0.695
University degree (no university as reference)	−0.04	0.29	0.02	0.96 (0.55–1.68)	0.893
Employed (unemployed as reference)	0.65	0.37	3.08	1.92 (0.93–3.97)	0.079
Sedentary work (yes)	−0.02	0.28	0.01	0.98 (0.57–1.68)	0.929
Caring for a child with a disability (yes)	−0.15	0.75	0.04	0.86 (0.20–3.75)	0.843
Caring for a child with a chronic disease (yes)	−0.73	0.87	0.70	0.48 (0.09–2.64)	0.402
Caring for an ill adult family member (yes)	−0.72	0.62	1.35	0.49 (0.14–1.64)	0.245
Caring for an older person (yes)	−0.66	0.46	2.07	0.52 (0.21–1.27)	0.151
Cigarette smoking (yes)	−0.22	0.25	0.76	0.80 (0.49–1.32)	0.384
E-cigarette use (yes)	0.05	0.28	0.03	1.05 (0.61–1.83)	0.855
Alcohol consumption (yes)	−0.31	0.25	1.58	0.73 (0.45–1.19)	0.208
Attitude toward a healthy lifestyle (score)	−0.06	0.04	2.61	0.94 (0.88–1.01)	0.106
Number of daily meals	−0.23	0.12	3.68	0.80 (0.63–1.00)	0.055
Sitting time (hours per day)	0.03	0.04	0.61	1.04 (0.95–1.13)	0.436
Sleep duration (hours per day)	0.27	0.11	5.99	1.31 (1.05–1.62)	0.014
BMI	0.08	0.02	19.44	1.08 (1.04–1.12)	<0.001

Note: β = beta coefficient; S.E. = standard error; Wald = Wald test; OR (95% CI) = Odds ratio with 95% confidence interval; *p* = *p* value; BMI = Body Mass Index.

## Data Availability

Data are available from the corresponding author upon reasonable request.

## Supplementary Materials

The following supporting information can be downloaded at: https://www.mdpi.com/article/10.3390/nu18030500/s1, Supplementary Table S1: Attitudes Toward A Healthy Lifestyle Questionnaire; Supplementary Table S2: Sociodemographic characteristics of the study participants (N = 570); Supplementary Table S3: Partial Spearman correlations between attitude toward a healthy lifestyle and lifestyle behaviors in the total sample (N = 570).

## Abbreviations

The following abbreviations are used in this manuscript:

NCDs	Non-communicable diseases
WHO	World Health Organization
BMI	Body Mass Index
CVI	Content Validity Index
I-CVI	Item-level Content Validity Index
S-CVI	Scale-level Content Validity Index
IQR	Interquartile range
β	beta coefficient
OR	Odds ratio
CI	Confidence interval
Mdn	Median
N	Number of participants
*p*	*p* value
S.E.	Standard error
Wald	Wald test
