# Supplementary material for: Association Between Attitude Toward a Healthy Lifestyle, Lifestyle Behaviors, Sociodemographic Characteristics, and Body Mass Index: A Cross-Sectional Study
Source: Nutrients. 2026 Feb 2;18(3):500. doi: 10.3390/nu18030500 (PMC12899998; doi:10.3390/nu18030500)
Supplement: Supplementary file 1 [file nutrients-18-00500-s001.zip › nutrients-4115537-supplementary.pdf]

Supplementary Table S1. Attitudes Toward A Healthy Lifestyle Questionnaire						
1.	I find it difficult to maintain healthy habits due to everyday obligations.	1	2	3	4	5
2.	I often find it easier to trust medications than lifestyle changes.	1	2	3	4	5
3.	My personal lifestyle has a strong impact on my health and well-being.	1	2	3	4	5
4.	I sometimes think that the impact of stress on health is overstated.	1	2	3	4	5
5.	I feel motivated to change my lifestyle habits when I realize that something is harmful to me.	1	2	3	4	5
6.	Sleep is a priority for me compared to other obligations.	1	2	3	4	5
7.	I have difficulty maintaining a balanced diet and regularly preparing home-cooked meals.	1	2	3	4	5
8.	Maintaining a healthy lifestyle requires more effort than I am sometimes willing to invest.	1	2	3	4	5
9.	I am regularly physically active for at least two hours per day (this includes all forms of physical activity).	1	2	3	4	5
10.	I find it difficult to assess how much interpersonal relationships truly affect health.	1	2	3	4	5
11.	I try to prevent stress in everyday life from affecting my health.	1	2	3	4	5
12.	The environment in which I live makes it difficult to maintain a healthy lifestyle.	1	2	3	4	5
13.	Support from family and friends helps me maintain healthy habits.	1	2	3	4	5
14	I sometimes neglect interpersonal relationships because I focus on obligations.	1	2	3	4	5
15.	I avoid harmful habits, such as smoking and excessive alcohol consumption.	1	2	3	4	5

Note: Attitudes toward a Healthy Lifestyle Questionnaire, assess attitudes toward the healthy habits and motivation and six core pillars of lifestyle medicine: Nutrition (items 7, 8); Physical activity (item 9); Sleep (item 6); Stress (items 4, 11); Interpersonal relationships (items 10, 13, 14); Harmful habits, such as smoking and alcohol (items 2, 15); and Healthy habits and motivation (items 1, 3, 5, 12).
Items were rated on a 5-point Likert scale (1 = Strongly disagree, 2 = Mostly disagree, 3 = Neither agree nor disagree, 4 = Mostly agree, 5 = Strongly agree).
Reverse-coded items (1, 2, 4, 7, 8, 10, 12, 14) were scored inversely.
Total scores were calculated by summing all items, with higher scores indicating more positive attitudes toward healthy lifestyle behaviours (possible range: 15–75).

Supplementary Table S2. Sociodemographic characteristics of the study participants (N = 570).	
Age (years), Mdn (IQR)	44.0 (15.0)
Age group (years), N (%)	
≤ 20	40 (7.0)
21–30	118 (20.7)
31–45	232 (40.7)
46–60	156 (27.4)
61–75	24 (4.2)
Sex, N (%)	
Female	478 (83.9)
Male	92 (16.1)
Place of residence, N (%)	
Urban	430 (75.4)
Rural	140 (24.6)
Marital status, N (%)	
Married / In a domestic partnership	352 (61.8)
In a relationship	75 (13.2)
Divorced	31 (5.4)
Widowed	8 (1.4)
Single	104 (18.1)
Having children (N, %)	
Yes	353 (61.9)
No	217 (38.1)
Number of children, Mdn (IQR)	2.0 (1.0)
Education level, N (%)	
Primary school	5 (0.9)
High school	174 (30.5)
Bachelor's degree	119 (20.9)
Master's degree	248 (43.5)
PhD	24 (4.2)
Employment status, N (%)	
Unemployed	32 (5.6)
Employed	472 (82.8)
Student	54 (9.5)
Retired	12 (2.1)
Type of work, N (%)	
Sedentary, minimal activity, minimal interpersonal contact	34 (6.0)
Sedentary, minimal activity, high interpersonal contact	210 (36.8)
Occasional activity, moderate interpersonal contact	97 (17.0)
Constant physical work, moderate interpersonal contact	30 (5.3)
Occasional physical work, minimal interpersonal contact	11 (1.9)
Intensive physical work, constant interpersonal contact	125 (21.9)
Currently not working	63 (11.1)
Caring for a child with a disability, N (%)	
Yes	23 (4.0)
No	547 (96.0)
Caring for a child with a chronic disease, N (%)	
Yes	22 (3.9)
No	548 (96.1)
Caring for an ill adult family member, N (%)	
Yes	54 (9.5)
No	516 (90.5)
Caring for an older person, N (%)	
Yes	69 (12.1)
No	501 (87.9)

Note: N = number of participants; Mdn = median; IQR = interquartile range.

Supplementary Table S3. Partial Spearman correlations between attitude toward a healthy lifestyle and lifestyle behaviors in the total sample (N = 570)*

		Sitting time	Sleep duration	BMI	Attitude toward a healthy lifestyle
Number of daily meals	R	-0.06	0.10	0.04	0.20
	p	0.188	0.016	0.344	< 0.001
Sitting time	R		0.05	0.02	-0.12
	p		0.276	0.633	0.007
Sleep duration	R			-0.03	0.25
	p			0.476	< 0.001
Body Mass Index	R				-0.22
	p				< 0.001

Note: *Partial Spearman correlations were calculated adjusting for age, sex, employment status, educational level, and type of work.
